# Disruption of gut barrier integrity and host–microbiome interactions underlie MASLD severity in patients with type-2 diabetes mellitus

**DOI:** 10.1080/19490976.2024.2304157

**Published:** 2024-01-18

**Authors:** R. Forlano, L. Martinez-Gili, P. Takis, J. Miguens-Blanco, T. Liu, E. Triantafyllou, C. Skinner, R Loomba, M. Thursz, J. R. Marchesi, B.H. Mullish, P. Manousou

**Affiliations:** a Liver unit/Division of Digestive Diseases, Department of Metabolism, Digestion and Reproduction, Faculty of Medicine, Imperial College London, London, UK; b Section of Bioinformatics, Division of Systems Medicine, Department of Metabolism, Digestion and Reproduction, Faculty of Medicine, Imperial College London, London, UK; c National Phenome Centre, Imperial College London, London, UK; d NAFLD Research Center, Division of Gastroenterology. University of California at San Diego, La Jolla, CA, USA

**Keywords:** Microbiome, gut permeability, fibrosis, type-2 diabetes mellitus, MASLD, metabolomics

## Abstract

Aberration of the “gut-liver axis” contributes to the development and progression of metabolic dysfunction-associated steatotic liver disease (MASLD). Here, we use multi-omics to analyze the gut microbiota composition and metabolic profile of patients with type-2 diabetes mellitus (T2DM). T2DM patients were screened for liver disease by blood tests, ultrasound, and liver stiffness measurements. Stool microbiota was analyzed by 16S rRNA gene sequencing; metabolomic profiling by Nuclear Magnetic Resonance spectroscopy and Ultra-High Performance-Mass Spectrometry. Microbiome and metabolic signatures were analyzed in the whole cohort and in matched subsets to identify signatures specific for steatosis (MASLD±) or fibrosis (Fibrosis±). Gut permeability was assessed *in-vitro* using monolayers of MDCK cells and trans-epithelial electric resistance (TEER). Cytokine profile was assessed in serum and stools.

Overall, 285 patients were enrolled: 255 serum, 252 urine and 97 stool samples were analyzed. *Anaeroplasma* and *Escherichia/Shigella* ASVs were higher, while *Butyricicoccus* ASVs were lower in those with normal liver. In MASLD±, *Butyricicoccus* ASV was significantly higher in those with steatosis. In the Fibrosis±, *Butyricicoccus* ASV was significantly lower in those with fibrosis. Glycochenodeoxycholic acid-3-sulfate (G-UDCA-3S) appeared to be higher in MASLD with fibrosis. Fecal water from patients with MASLD and fibrosis caused the greatest drop in the TEER vs those with normal liver; this was reversed with protease inhibitors. Finally, fecal IL-13 was lower in MASLD with fibrosis. We identified microbiome signatures which were specific for steatosis and fibrosis and independent of other metabolic risk factors. Moreover, we conclude that protease-related gut permeability plays a role in those MASLD patients with fibrosis, and that disease progression is linked to a gut-liver axis which is at least partially independent of T2DM.

## 1. Introduction

Metabolic dysfunction-associated steatotic liver disease (MASLD) represents a leading cause of liver disease worldwide^1^ and is now the fastest growing indication for liver transplantation.^2^ Due to the dramatic increase in the global incidence of metabolic risk factors and the aging population, the burden from advanced liver disease from MASLD is expected to double by 2030.^3^ Notably, metabolic risk factors in children and adolescents constitute one of the biggest threats to global health in the coming decades. Among other metabolic risk factors, type-2 diabetes mellitus (T2DM) is associated with higher prevalence of advanced fibrosis, progression to cirrhosis and development of liver cancer in these patients. Several clinical and pre-clinical studies have demonstrated that T2DM can contribute to liver damage through several mechanisms, including enhanced *de novo* lipogenesis,^4^ hepatotoxicity^5^ and oxidative stress.^6^

Over the last decade, a growing body of research has supported the association between a perturbation of the gut microbiome and metabolic diseases, including MASLD. Aberration of the complex crosstalk between the intestine and the liver, often called the “gut-liver axis”, has been suggested to be a contributory factor in the development and progression of liver disease in patients with MASLD.^7,8^

Initial clues to a microbiome contribution to MASLD pathophysiology were derived from studies associating metabolic-dysfunction associated steato-hepatitis (MASH) with bacterial overgrowth in the human small intestine.^9^ Furthermore, changes in gut permeability,^10^ as well as an increased intestinal inflammation,^11^ may drive the translocation of bacterial products from the intestinal lumen to the liver, leading to liver inflammation and injury.^12^ Hence, gut permeability may represent a key modulator in the crosstalk between host and microbiome, in MASLD and other metabolic disorders.^10^ Some studies have shown an association between fibrosis stage and metagenomic signature of gut microbiome in MASLD.^13,14^ Interestingly, several studies have supported the administration of fecal microbiota transplant (FMT) as a possible therapy in patients with MASLD. A recent study found that when obese males with metabolic syndrome received an FMT from lean male donors, they showed a transient, but significant improvement in insulin resistance and in butyrate-producing intestinal microbiota.^15^ Interestingly, a combination of FMT with diet changes provided better glycemic control and other metabolic parameters in patients with T2DM.^16^

Nevertheless, a major confounder in exploring the contribution of the gut microbiome to MASLD in humans relates to insulin resistance-associated metabolic comorbidities,^5^ as these are also involved in the pathogenesis and progression of MASLD, and are themselves associated with distinctive perturbations of gut microbiome composition and function.^7^ As such, disentangling the impact of metabolic risk factors on the results from previous studies is challenging. Moreover, there is a high inter-study variability on how the severity of MASLD is assessed, often focusing on degree of steatosis, or simple steatosis *versus* MASH, rather than a more clinically relevant measure such as fibrosis severity.^17,18^

In this study, we used a multi-omics approach to analyze the bacterial composition and metabolic profile of a prospective cohort of patients with type-2 diabetes mellitus (T2DM) screened for liver disease and fibrosis in the community.^19^

## 2. Results

### 2.1. Characteristics of the study population

A total of 285 patients were enrolled in the study: clinical features are shown in Table 1. 255 serum, 252 urine and 97 stool samples were available for analysis. Samples from patients with other causes of liver diseases (e.g., excessive alcohol consumption, chronic hepatitis B, etc) were excluded from this analysis.

**Table 1.** Characteristics of the study population and differences between patients with and without MASLD. The table shows the differences between patients with (*n* = 183) and without (*n* = 73) MASLD in the whole study population (*n* = 284). Variables are expressed as median and IQR or relative percentages. * p-value refers to differences between patients with MASLD and normal liver.

	Study population N = 284	MASLD N = 182	Normal liver N = 73	
	Median (IQR)	Median (IQR)	Median (IQR)	P value*
Age, *years*	59 (59–66)	60 (54–66)	59 (53–65)	0.83
Waist circum, *cm*	107 (107–116)	108 (101–118)	98 (92–106)	0.0001
Hip circum, *cm*	110 (102–119)	112 (105–122)	103 (98–108)	0.0001
BMI, *kg/m*^*2*^	30.8 (26.9–34.4)	31.4 (28.4–35.8)	26.9 (24.8–30.3)	0.0001
PLT, *x 10*^*9*^*/µL*	250 (202–290)	245 (212–287)	249 (206–298)	0.88
ALT, *IU/L*	35 (22–45)	34 (23–49)	24 (18–28)	0.0001
AST, *IU/L*	31 (22–35)	28 (23–37)	24 (19–27)	0.0001
GGT, *IU/L*	47 (19–50)	32 (22–52)	19 (17–27)	0.0001
ALP, *IU/L*	88 (70–103)	84 (72–105)	85 (63–99)	0.7
Albumin, *g/L*	40 (39–42)	41 (39–42)	40 (39–42)	0.83
Bilirubin, *µmol/L*	10.6 (6–12)	9 (6–12)	8 (6–14)	0.55
Total Cholesterol, *mmol/l*	4.1 (3.5–4.7)	4.1 (3.4–4.7)	4 (3.6–4.5)	0.58
TRG, *mmol/l*	2.3 (1.02–2.08)	1.4 (1.07–2.1)	1.2 (0.98–1.5)	0.25
HDL, *mmol/l*	1.1 (0.9–1.3)	1.1 (0.9–1.2)	1.16 (1.06–1.39)	0.25
LDL, *mmol/l*	2.3 (1.6–2.7)	2.2 (1.6–2.8)	2.1 (1.7–2.6)	0.68
Ferritin, *ng/ml*	124 (43–155)	82 (39–140)	70 (28–178)	0.91
Diabetes characteristics				
Fasting glucose, *mmol/l*	7.9 (5.5)	7.4 (5.6–10.2)	6.2 (4.8–7.8)	0.001
HbA1c, *mmol/mol*	60 (49–70)	60 (50–74)	55 (48–61)	0.0001
Insulin, *µU/ml*	24 (8.1–26.5)	15.3 (9.8–28.2)	7.2 (5.8–12.2)	0.028
HOMA index	8 (1.9–8.95)	4.6 (2.2–10.3)	2.1 (1.35–4.8)	0.0001
Duration DM, *years*	11 (4–16)	10 (3–16)	13 (7–16)	0.16
	N (%)	N (%)	N (%)	P value*
Diet controlled	39 (13)	25 (13)	13 (18)	0.11
On oral agents	227 (79)	170 (91)	55 (75)	0.16
On GLP-1RA	37 (13)	31 (16)	6 (8)	0.08
On insulin	74 (25)	51 (28)	23 (31)	0.18
Diabetic complications	45 (16)	26 (14)	15 (21)	0.82
Ethnic background and comorbidities				
	N (%)	N (%)	N (%)	P value*
Male gender	160 (53)	104 (56)	34 (45)	0.07
White, Caucasian	102 (32)	64 (34)	15 (20)	0.02
White, Hispanic	6 (2)	3 (1)	2 (2)	0.43
Black African, Afro-Caribbean	33 (12)	22 (12)	10 (13)	0.41
Arab	74 (28)	52 (28)	20 (26)	0.52
South Asian	47 (17)	31 (17)	16 (21)	0.2
East Asian	24 (8)	14 (7)	10 (13)	0.09
Hypertension	191 (67)	120 (64)	50 (66)	0.32
Dyslipidaemia	148 (52)	98 (53)	39 (52)	0.51
Psychiatric disorder	41 (15)	27 (14)	11 (14)	0.53
Previous ACE inhibitor	28 (10)	16 (8)	11 (14)	0.98
On statin	214 (75)	138 (74)	57 (76)	0.31

*Abbreviations: IQR: interquartile range, BMI: Body mass index, PLT: platelet, ALT: alanine aminotransferase, AST: aspartate aminotransferase, GGT: gamma-glutamyl transferase, ALP: alkaline phosphatase, TRG: triglycerides, HDL: high density lipoprotein, LDL: low density lipoprotein, HbA1c: glycated haemoglobin, GLP-1RA: glucagon like peptide-1 receptor agonist.*

When compared to those with MASLD and normal LSM (*n* = 136, 17%), patients with significant fibrosis (LSM ≥8.1 kPa) (*n* = 50, 6%) presented with higher body mass index (BMI) (36.8 vs 30.3 kg/m^2^, *p* = 0.0001), and larger hip (123 vs 110 cm, *p* = 0.0001) and waist circumferences (120 vs 105 cm, *p* = 0.0001). In terms of metabolic control, patients with MASLD and significant fibrosis had poorer diabetic control: higher median HbA1c (71 vs 59 mmol/mol, *p* = 0.0001), fasting glucose (9.4 vs 6.7 mmol/l, *p* = 0.001), insulin level (21 vs 12.4 µU/ml, *p* = 0.001) and HOMA-IR index (8.1 vs 3.3, *p* = 0.001). Due to the observed correlation between liver fibrosis and T2DM (Table 1 and **Supplementary Figure S1**), we sought to understand metabolic and bacterial differences in the whole cohort, but also among patients matched by metabolic risk factors (see Methods).

### 2.2. Distinct taxonomic compositions differentiate the gut microbiome of diabetics with and without MASLD, and based upon MASLD severity

Overall, gut bacterial ecology and composition was analyzed in a subset of 97 patients (20 without liver disease; 58 MASLD with no significant fibrosis; 19 MASLD with significant fibrosis) for which fecal samples were available.

In the unmatched analysis including all sampled individuals, there was no difference in terms of alpha diversity richness or evenness measures between groups. Similarly, beta diversity assessed with the Aitchison distance did not show a visible clustering among groups (Supplementary Figure S2). With regards to taxonomy, abundance of the *Bacteroidota* and *Firmicutes* (now named *Bacillota*) phyla was lower in MASLD with fibrosis patients, but similar in MASLD and patients without liver disease. *Proteobacteria* (now named *Pseudomonadota*) were higher in patients without liver disease, and similar in the other two groups (Supplementary Figure S2). At an ASV level, *Anaeroplasma* and *Escherichia/Shigella* ASV were lower, while *Butyricicoccus* ASV higher in MASLD with fibrosis (Supplementary Figure S2).

**Figure 1.**
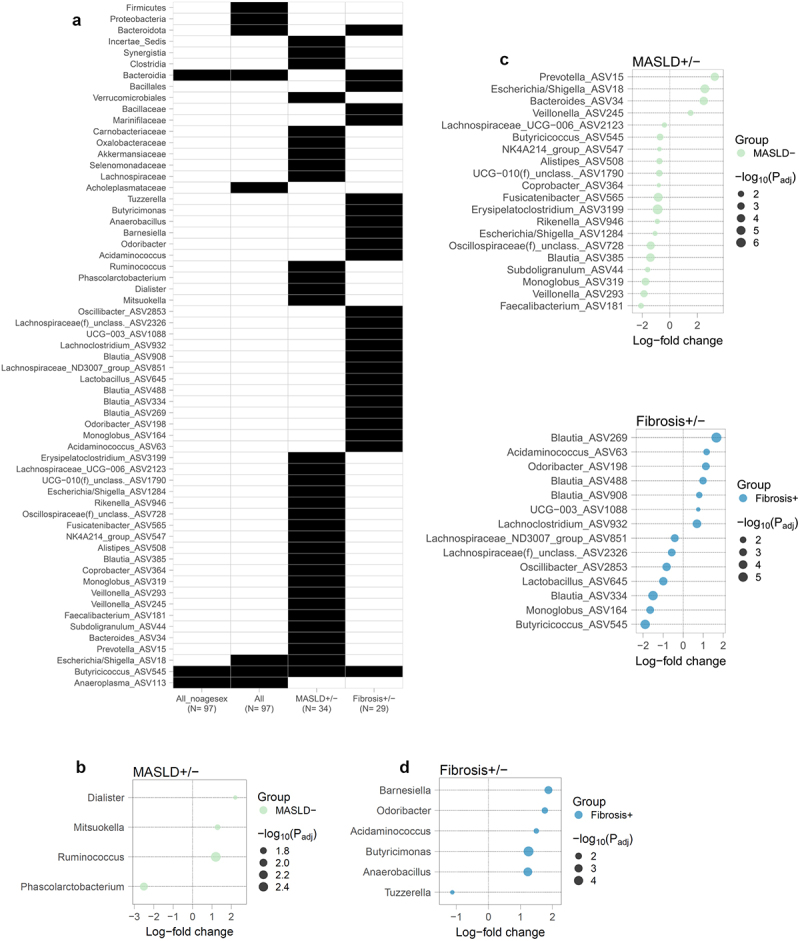
a) Taxonomic features significant across unmatched and matched comparisons. All_noagesex comparison did not include age and sex as covariates in the model. b) Log-fold change of differentially abundant genera shown in panel A, determined with ANCOMBC in MASLD± (left) and Fibrosis± (right) matched analyses. c) Log-fold change of differentially abundant ASVs shown in panel A, determined with ANCOMBC in MASLD± (top) and Fibrosis± (bottom) matched analyses.

In the matched MASLD± sub-analysis (see **Methods**), there was no difference in terms of alpha diversity between paired patients with and without MASLD. With regards to taxonomy, no phylum differences were found between those with MASLD and those without. At the genus level, a lower abundance of *Mitsuokella*, *Dialister* and *Ruminococcus* and higher abundance of *Phascolarctobacterium* were specific for the presence of steatosis (Figure 1b), and 20 ASVs were also found differently abundant (Figure 1c and Supplementary Table S2).

In the Fibrosis± sub-analysis, there was a higher Simpson, inverse Simpson and Shannon evenness in those with fibrosis, compared to those without (Supplementary Figure S2). With regards to taxonomy, only the *Bacteroidota* phylum was still significantly lower in those with fibrosis compared to those without (β=-0.83; 95% CI [−1.23,-0.44]; Figure 1a and Supplementary Table S3). At the genus level, higher abundance of *Acidaminococcus*, *Odoribacter*, *Barnesiella*, *Anaerobacillus*, *Butyricimonas* and lower abundance of *Tuzzerella*, was specific for the presence of fibrosis (Figure 1b). Finally, *Butyricicoccus* ASV was also significantly lower in those with fibrosis compared to those without (Figure 1c).

### 2.3. Host-microbiome co-metabolites are different in different disease stages

In the unmatched analysis including all individuals, those with MASLD and fibrosis showed higher serum levels of acetone, glucose, glutamic acid, phenylalanine, lactic acid and citric acid (**Supplementary Figure S3**). In terms of urinary metabolites, alanine was significantly higher in those with MASLD compared to those without liver disease (β= −0.28; 95% CI[−0.46,-0.1]; normal liver with respect to MASLD). There was no difference in the fecal metabolite profiles between groups.

In the matched MASLD ± sub-analysis, those with MASLD showed lower serum levels of asparagine, proline, picolinic acid and citrulline compared to those without (asparagine: β = 0.07; 95% CI [0.024,0.11], proline: β = 0.064; 95% CI [0.016,0.11], picolinic acid: β = 0.22; 95%CI [0.03,0.4], citrulline: β = 0.24; 95%CI [0.12,0.37]; normal liver with respect to MASLD). There were no differences in the urinary and fecal metabolite profiles between groups.

In the fibrosis ± sub-analysis, those with fibrosis showed higher serum levels of glutamate and acetone compared to those without fibrosis. In addition, serum lysine was higher (β = 0.09; 95% CI [0.036,0.14]) and valine lower (β= −0.058; 95% CI [−0.091,-0.24]) in those with fibrosis, compared to those without. Regarding fecal metabolites, glycine was significantly lower in those with fibrosis, compared to those without (β= −0.22; 95% CI[−0.38,-0.06]).

Finally, no differences were found in serum lipoproteins in either the unmatched or the matched analyses.

### 2.4. Steatosis and fibrosis in MASLD both influence characteristic serum bile acid signatures

In the unmatched analysis including all individuals, those with MASLD and fibrosis showed higher levels of serum primary BAs taurocholic acid (TCA) and glycochenodeoxycholic acid-3-sulfate (G-CDCA-3S), and its secondary 7β-OH bile acid glycoursodeoxycholic acid-3-sulfate (G-UDCA-3S) compared to those with MASLD without fibrosis (Supplementary Figure S3).

**Figure 2.**
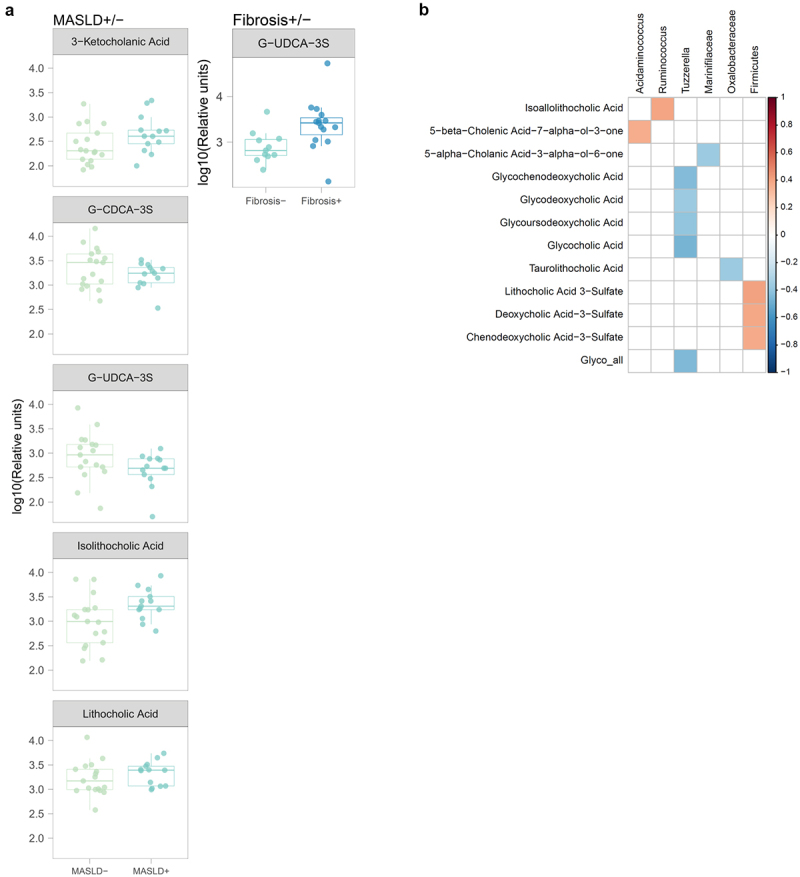
a) relative intensities of differentially abundant bile acids in the MASLD± (left; *n* = 17 MASLD-; *n* = 13 MASLD+) and Fibrosis± analyses (right; *n* = 11 fibrosis-; *n* = 15 Fibrosis+). b) Pearson correlation heatmap of fecal bile acids relative intensities and significant taxonomic features found in the cohort. *P*-values were adjusted using Benjamini–Hochberg, with a 10% false discovery rate threshold (Padj <0.1). Only significant correlations with an absolute coefficient value equal or bigger than 0.2 are shown. *n* = 20 no liver disease; *n* = 58 MASLD without fibrosis; *n* = 19 MASLD with fibrosis.

In the MASLD ± sub-analysis, those with MASLD presented with significantly higher levels of serum isolithocholic acid, 3-ketocholanic acid, and lithocholic acid compared to those without MASLD. Moreover, those with MASLD had significantly lower levels of G-CDCA-3S, and G-UDCA-3S (Figure 2a).

In the fibrosis ± sub-analysis, only G-UDCA-3S remained significantly higher in those with fibrosis compared to those without (Figure 2a).

In contrast to the bile acid signature found in serum, no differences in fecal bile acids were found in either the unmatched or the matched analyses. Nevertheless, a correlation analysis was performed between fecal bile acids and the differently abundant metataxonomic data. Overall, the summed intensities of glycine-conjugated bile acids were negatively correlated with abundance of *Tuzerella* genus. Moreover, sulfated bile acids (deoxycholic acid-3-sulfate, chenodeoxycholic acid-3-sulfate and lithocholic acid-3-sulfate) were elevated in fecal samples of patients enriched with *Firmicutes* (Figure 2b).

### 2.5. MASLD with significant fibrosis is characterised by gut luminal pro-inflammatory status

Cytokines profiles were tested in a total of 138 serum samples: 41 (30%) patients without liver disease, 60 (43%) with MASLD, and 37 (27%) with MASLD and significant fibrosis. Higher levels of pro-inflammatory cytokines IFNγ, IL-6, IL-8 and TNFα were found in MASLD with fibrosis patients compared with MASLD without fibrosis. There were no differences between no liver disease and MASLD without fibrosis patients (Supplementary Figure S3).

A total of 76 fecal samples were tested for cytokine profile: 18 (24%) without liver disease, 38 (50%) MASLD without fibrosis, 20 (26%) MASLD with fibrosis. Only IL-13 was significantly lower in MASLD with fibrosis patients, when compared to both unmatched and matched MASLD patients without fibrosis (β= −0.24; 95%CI [−0.41,-0.085]).

Fatty acid binding protein 2 (FABP-2) and plasminogen activator inhibitor type I (PAI-1 or SERPINE1) were analyzed as markers of gut permeability and intestinal damage. Median serum concentration for FABP-2 was 2662.8 (1342.2–3595.3) pg/ml in those with normal liver, 1608.5 (992.4–2782.7) pg/ml in those with MASLD without fibrosis, 3112.2 (1987.5–3900.3) pg/ml in those with MASLD with fibrosis; overall, there was no difference in serum FABP-2 nor PAI-1 across study groups.

### 2.6. MASLD with significant fibrosis is characterized by protease-mediated gut barrier dysfunction

Given the proinflammatory profile of the patients, we went on to explore mechanisms of gut barrier dysfunction in these patients *in vitro*. Fecal water (FW) from 12 patients enrolled in the study (5 without liver disease, 3 with simple MASLD and 4 with MASLD and fibrosis) was incubated with a monolayer of MDCK cells, and monolayer integrity was estimated using trans-epithelial electric resistance (TEER). Clinical characteristics of the patients whose samples were used for this analysis are shown in Supplementary table S2.

**Figure 3.**
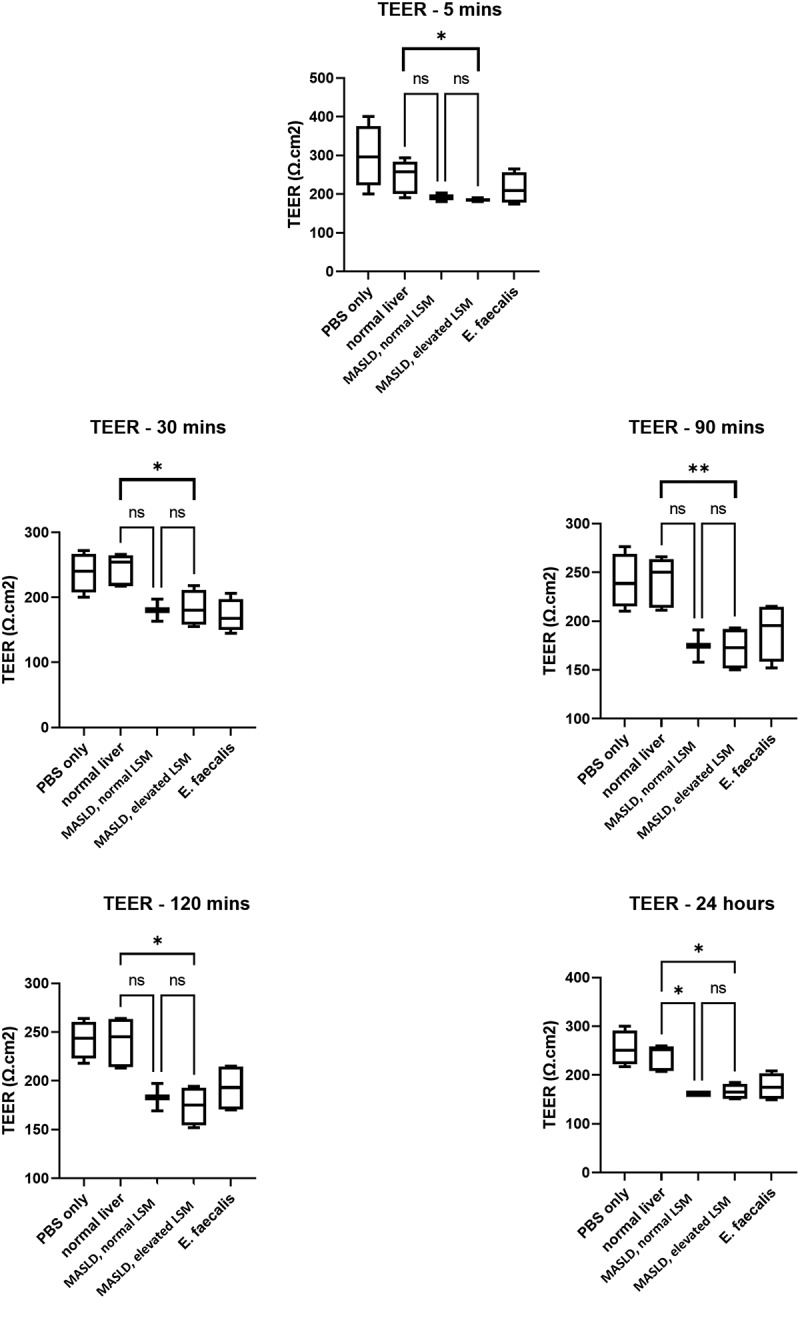
Effect of uninhibited fecal water, positive and negative controls on TEER of MDCK monolayers. The box plot illustrates median values of triplicate measurements of TEER and corresponding 95%CI and difference between study groups: normal liver (*n* = 5), MASLD with normal LSM (*n* = 3) and MASLD with elevated LSM (*n* = 4). *abbreviations: 95%CI: 95% confidence interval, TEER: trans-epithelium electric resistance.*

Overall, the samples from patients with MASLD and fibrosis caused the greatest change in the TEER compared to those with normal liver. Specifically, when comparing monolayers incubated with fecal extracts from patients with MASLD and fibrosis vs samples from those with normal liver, TEER was 185 vs 258 Ωcm^2^ (*p* = 0.04) five minutes after fecal extract exposure, 132 vs 247 Ωcm^2^ (*p* = 0.032) at 30 mins, 172 vs 250 Ωcm^2^ (*p* = 0.037) at 90 mins, 175 vs 245 Ωcm^2^ (*p* = 0.026) at 120 mins and 164 vs 252 Ωcm^2^ (*p* = 0.002) at 24 hours (Figure 3).

To explore whether bacterial proteases were associated with the decrease in the monolayer resistance, a commercial cocktail of bacterial protease inhibitors was added to FW (inhibited FW). Inhibited FW caused a significant lower decrease in TEER compared to uninhibited FW in those with MASLD without fibrosis, and in those with MASLD with fibrosis. Interestingly, there was no significant change in TEER when using inhibited or uninhibited FW from patients with normal liver (Supplementary Material).

We explored whether TEER correlated with clinical parameters. An overall median TEER was calculated as the median of the TEER measurements from 5 minutes to 24 hours. In terms of association with clinical features, there was a strong, inverse linear relationship between TEER and body mass index (BMI, Rho = −0.78, *p*  = 0.01 and R2 = 0.43, *p*  = 0.029) and between overall TEER and waist circumference (Rho = −0.69, *p*  = 0.026 and R2 = 0.51, *p*  = 0.019). Moreover, there was a strong, inverse linear relationship between TEER and AST (Rho = −0.65, *p*  = 0.03 and R2 = 0.402, *p*  = 0.036) and between TEER and LSM (Rho = −0.88, *p*  = 0.0001 and R2 = 0.55, *p*  = 0.009). Nevertheless, there was no relationship with HbA1c (*p*  = 0.069), HOMA index (*p*  = 0.88) or ALT (*p*  = 0.07).

## 3. Discussion

Over recent years, there has been an increasing number of studies exploring changes in the microbiome and its association with MASLD. Notably, the majority of studies have focused on comparing healthy controls *vs* patients with MASH or comparing different grades of steatosis,^20^ with only a relatively small body of evidence exploring specific changes in gut microbiota with regards to liver fibrosis in MASLD, despite this being the main prognostic factor for clinical outcomes in these patients.^21^ Even where such studies do exist, few controls for key confounders of other metabolic syndrome features, and a focus on tertiary center cohorts, may skew generalizability of interpretation.^22^ Specifically, T2DM carries independent changes in the gut microbiome, with several studies suggesting declined bacterial diversity and specific microbial changes in diabetics.^23^ As such, disentangling the impact of single metabolic risk factors on microbiome signatures in MASLD patients is challenging.^7^ Furthermore, many studies have assessed the severity of liver disease based on abnormal liver enzymes, which are poor markers of disease stage and severity.^24^ As many noninvasive techniques are now available in clinical practice,^25^ they should be leveraged to stratify patients accordingly. Finally, while many MASLD studies have focused on gut microbiome composition, few have explored aspects of gut microbiome function and host-microbiome crosstalk by combining other systems biology techniques such as metabolomics. Our distinctive study design and consideration of metabolic confounders allowed us to directly explore the association between the gut microbiome and MASLD severity.

Previous studies have shown a higher abundance of *Proteobacteria* and *Firmicutes* (now named *Pseudomonadota* and *Bacillota*, respectively) as well as a reduction in *Bacteroidota* and *Prevotellaceae* in patients with MASLD compared to healthy controls.^20,22^ However, as metabolic factors such as glycemic control, BMI, waist circumference, are part of the metabolic syndrome and MASLD spectrum, it proves challenging to deconvolute their individual relationship with microbiome composition. In this study, when patients with similar metabolic characteristics were compared, we found several microbial features specifically associated with steatosis or fibrosis in our cohort (Figure 1b). On a phylum level, patients with MASLD and fibrosis were characterized by a lower abundance of *Bacteroidota*, and neither *Firmicutes* nor *Proteobacteria* were specific of liver steatosis or fibrosis. Previous studies had suggested that *Firmicutes* to *Bacteroidota* ratio may distinguish those with MASLD from healthy controls.^22^ However, our results suggest that *Firmicutes* abundance might be influenced by metabolic status rather than being specific of liver disease in these patients. A *Butyricicoccus* ASV was significantly lower in those with normal liver and in those with MASLD fibrosis compared to those with steatosis only. Given that *Butyricicoccus* is a probiotic, butyrate-producing genus, higher abundance of this ASV in patients with steatosis might be a compensatory mechanism.

In this study, patients with MASLD had higher levels of serum lithocholic acid (LCA) compared to those with normal liver. LCA may exert a hepatotoxic effect^26^ and its serum concentration may be modulated by specific changes in the gut microbiome, such as a reduction in *Bacteroides* spp.^27^ LCA is also the precursor of isolithocholic acid (isoLCA), where the 3α-OH has been modified by bacterial 3α/β-hydroxysteroid dehydrogenases (3α/β-HSDH). Interestingly, isoLCA was also higher in MASLD compared to individuals without MASLD, and we found a positive correlation between *Ruminococcus* genus – lower in MASLD – and isoallolithocholic acid (isoalloLCA), an isoLCA epimer with a 5α-H group. Higher levels of serum G-UDCA-3S were specific for significant fibrosis due to MASLD, and this finding was independent of metabolic risk factors. To date, very little is known about G-UDCA-3S and its role in health or in disease. However, in the matched analyses we found that G-UDCA-3S was elevated in those with normal liver and in those with elevated fibrosis, but lower in those with MASLD without fibrosis, indicating that a decrease in G-UDCA-3S might relate to the etiology of steatosis. In addition, we found a negative correlation between *Tuzzerella* genus – lower in MASLD with fibrosis – and G-UDCA in feces, indicating that changes in this genus could be modulating UDCA levels. *Tuzzerella* abundance negatively correlated with overall summed intensities of glycine-conjugated BAs (Figure 2), and as such its lower abundance in the gut could be indicative of attenuated liver BA-conjugating function as well as fibrosis.

In this study, high serum glutamate and acetone, as well as lower valine, were specific for the presence of fibrosis in this population, independently of other metabolic risk factors. Of note, glutamic acid is a non-essential amino acid, derived mainly via the catabolism of glutamine (glutaminolysis) in the liver.^28^ Interestingly, a recent study from Du and colleagues has demonstrated that the liver isoform of glutaminase may be upregulated by the hepatic stellate cells, as they require glutaminolysis to satisfy their energetic demand.^29^ Similarly, higher levels of lysine, an essential amino acid which is mainly catabolised in the liver, were specifically associated with liver fibrosis in this study. Of note, previous studies have associated lower lysine levels with collagen disturbances, because of over-expression of the enzyme lysil oxidases.^30^ In pathological condition such as fibrogenesis, such an enzyme is overexpressed and promotes collagen cross-linking and stabilization against proteolytic degradation, maintaining hepatic stellate cells in an activated state.^31^ Taken together, these results suggest a possible role for glutamate and lysine in predicting the presence of fibrosis in this population.

Microbiome perturbations may also compromise the intestinal tight junctions, increasing gut permeability and translocation of bacterial products.^32^ Overall, it has been demonstrated that small intestinal permeability increases with the degree of hepatic steatosis, while the association with severity of liver disease remains unexplored.^33,34^ Here, we set up a model *in vitro* to replicate the gut barrier, based on monolayers of MDCK cells. Interestingly, fecal water from patients with fibrosis caused the greatest change in the TEER compared to those with normal liver, and this increase was partially inhibited by bacterial protease inhibitors. Moreover, increased permeability correlated with severity of liver disease (assessed by LSM) and with visceral obesity, but not with glycemic control. Nevertheless, in this study, FABP-2 levels did not differ among groups, perhaps suggesting good enterocyte function. In addition, we found that levels of IL-13, a cytokine exerting anti-inflammatory effect in different contexts,^35^ were lower in the stools of those with significant fibrosis, suggesting that a pro-inflammatory *milieu* in the gut may be associated with more severe disease and contribute to increased gut permeability.

This study presents several strengths. Firstly, the population included has been well phenotyped both clinically and in terms of ‘omics’ techniques used. Also, our statistical analysis framework allowed for the identification of factors associated with MASLD and severity of liver disease in the whole population, with important confounders included as well as on an independent level (i.e. matched subsets, controlled for metabolic confounders). Furthermore, our analysis focuses on liver fibrosis, which is the main prognostic factor in patients with MASLD. This work also presents some limitations. Firstly, the sub-analysis of matched subjects included a smaller number of patients compared to the whole cohort, which could signify that some metabolic features specific to steatosis or fibrosis could not be detected due to loss of power. Secondly, fecal samples were available for the microbiome analysis only for a subgroup of patients, introducing potential bias in the analysis. Thirdly, limited information was available on dietary patterns from these patients; thus, analysis against diet could not be included. Finally, liver fibrosis was assessed using noninvasive markers rather than histology. However, previous studies support the use of elastography in primary care studies for MASLD.^36,37^

In this study, we identified microbiome signatures which were specific for steatosis and fibrosis and independent of other metabolic risk factors. Moreover, our findings suggest that gut permeability plays a pathological role in those MASLD patients with fibrosis, and that disease progression is linked to a gut-liver axis which is at least partially independent of T2DM.

## 4. Patients and methods

### 4.1. Patient enrolment and screening procedures

Participants were recruited and assessed in the United Kingdom, in a West London National Health Service (NHS) primary care setting, as previously described.^19^ Briefly, consecutive patients with T2DM were screened for liver disease and fibrosis using blood tests, transient elastography (TE) and ultrasound scans. Liver stiffness measurements (LSM) and controlled attenuation parameter (CAP) scores were assessed after 4 hours of fasting. Hepatic steatosis was defined based on US scan and CAP scores as per previously published criteria,^38^ while significant fibrosis was defined as LSM ≥ 8.1 kPa.^25^ Medical history, alcohol consumption, dietary intake and anthropometric parameters were recorded for each patient during the screening visit. All the assessments were performed in the Liver and Anti-Viral unit, St Mary’s Hospital, Imperial College Healthcare NHS Trust (London, UK).

Patients’ recruitment was conducted in line with Good Clinical Practice and sample handling according to Human Tissue Act regulations. The study obtained full ethical approval from the Research Ethics Committee (REC approval 18/LO/1742, IRAS 251,274), sponsorship from Imperial College London, London, UK, and adoption from Clinical Research Network Portfolio.

### 4.2. Sample collection and storage

Urine and serum samples were collected on the day of the screening and after 4 hours of fasting. Faecal samples were transported cold (using ice packs) and stored as crude material at −80°C within 6 hours. Stools underwent collection within one week of the screening procedures. All samples were stored at −80°C and processed in the Hepatology Clinical research facility based at St. Mary’s Hospital Campus, Imperial College London, London, UK, consistent with standard protocols.^39–41^

### 4.3. Metataxonomics

Faecal bacterial composition was analyzed by 16S rRNA gene amplicon sequencing of the V1-V2 gene region, using an Illumina MiSeq instrument and paired-end chemistry, as previously described.^42^ Primers were trimmed, paired ends merged, and amplicon sequence variants (ASVs) identified with DADA2.^43^ Taxonomic assignments for each ASV were called using UTAX trained on the SILVA v132 ribosomal database. More details are provided in Supplementary Material. For statistical analyses, except for α-diversity calculations, features with a zero count in more than 90% of samples were discarded, left-censored zero values were imputed with cmultRepl from *zCompositions* R-package, using a geometric Bayesian multiplicative (GBM) replacement method, and data were centered-log-ratio (clr)-transformed.

### 4.4. Metabolomic profiling

All assays were carried out at the MRC-NIHR National Phenome Centre (https://phenomecentre.org)., Imperial College London.

#### 4.4.1. Ultra-high performance liquid chromatography-mass spectrometry bile acid profiling

Stool and serum sample preparation for ultra-high performance liquid chromatography-mass spectrometry (UHPLC-MS) was performed as previously described,^41^ and samples were analyzed using an Acquity UPLC instrument coupled to a Xevo G2 Q-ToF mass spectrometer (Waters, Elstree, UK) equipped with an electrospray ionization source operating in negative ion mode, as previously described.^39^ More details on bile acid profiling are provided in the **Supplementary material**.

#### 4.4.2. Tryptophan metabolites

Stool concentrations of tryptophan-related metabolites were determined using the previously described protocols.^44^ Features below the limit of detection (LOD) in more than 20% of study samples were discarded. The remaining zeros were imputed using impute. QRILC from the imputeLCMD R-package and features were log-transformed and mean-centered.

#### 4.4.3. Proton Nuclear Magnetic Resonance spectroscopy

Proton Nuclear Magnetic Resonance spectroscopy^1^H-NMR) analysis was performed on serum, urine and fecal water following Bruker IVDr standard protocols, as previously described.^45,46^ Statistical correlation spectroscopy (STOCSY) and small molecule enhancement spectroscopy (SMolESY)^47,48^ were used for the small sized metabolites identification and quantification respectively. Serum lipoproteins were quantified by Bruker IVDr platform.^49^

### 4.5. Statistical analysis

Unmatched datasets, using all samples available, were analyzed with or without adjusting for age and sex. However, since liver stiffness correlated strongly with glycemic control and with obesity in this population (Supplementary Figure S1), identifying the factors independently associated with MASLD and fibrosis was challenging. Patients with significant fibrosis were metabolically too different to be compared to those with no liver disease. As such, MASLD patients without significant fibrosis (i.e. evidence of steatosis based upon ultrasound and CAP, but TE < 8kPa) were used as the reference group in all regression models, so that only effect sizes with respect to MASLD without fibrosis could be formally calculated. To seek MASLD-specific associations, two sub-analyses were done. In the 1st sub-analysis (‘MASLD ±‘), MASLD patients without fibrosis were matched to patients without liver disease to assess the differences related to presence/absence of MASLD in metabolically similar T2DM patients. In the 2nd sub-analysis (‘Fibrosis ±‘), MASLD patients without fibrosis were matched to patients with MASLD and significant fibrosis to assess differences between the presence/absence of fibrosis in metabolically similar T2DM patients diagnosed with MASLD. More details on the sample matching, including the variables used, are provided in Supplementary Material.

#### 4.5.1. Metabolomics

Differential abundance analysis of metabolic features across groups was done in R by fitting the following linear mixed effects model with *lme4* R-package and custom scripts^50^

feature ~ group + age + sex + alcohol + PPI + smoking + insulin + metformin + (1| IMD)

Where *group* had three categories: MASLD without fibrosis (reference), no liver disease and MASLD with fibrosis, *age* was mean-centered and univariance-scaled, *alcohol* had three categories: “abstinent” (reference; 0 units/week), “moderate” (1–14 units/week) and “excess” (>14 units/week), *smoking* had three categories: “never” (reference), “former” and “current”, *PPI*, *metformin* and *insulin* had two categories each, indicating whether patients regularly took proton pump inhibitors (PPI), metformin or other insulin drugs, respectively. The index of multiple deprivation (IMD) was split into decile categories and added as random effect to adjust for variability from socio-economic factors among participants. Missing records in *insulin* (*N* = 9; 3.2%) were imputed with the most common category, for missing *IMD* (*N* = 1; 0.35%) the median was imputed, for missing alcohol units per week (*N* = 1; 0.35%) the mean value was imputed and missing BMI records (*N* = 3; 1%) were predicted by linear regression with hip and waist circumference and sex as explanatory variables using lm. Significance of *group* was determined using a likelihood ratio test (LRT) of a null model without *group* but keeping the rest of the covariables. *p-*values were adjusted (Padj) for multiple comparisons using Benjamini–Hochberg and features were considered significant if Padj < 0.1.

#### 4.5.2. Metataxonomics

Alpha-diversity measures were calculated with estimate_richness function from the phyloseq R-package^51^ and differences across groups assessed using the same mixed effects model as before (see 4.5.1. Metabolomics analysis).

Beta-diversity was inspected using Principal Component Analysis (PCA) on the clr-transformed ASV counts^52^ with the opls function from the ropls R-package.^53^

Differential abundance analysis of metataxonomics data was done using *ANCOMBC* R-package^54^ with the same model as before (see Metabolomics analysis), but with *IMD* as a fixed effect. Identification of structural zeros was set to false, as we previously showed a high false positive rate when determining zeros in less than 50 samples.^55^ *P*-values were adjusted using the Holm method, with Padj < 0.05 considered significant.

Correlation heatmap was produced using corr.test function from the psych R-package and corrplot R-package. *p* values were adjusted with Benjamini–Hochberg method and a 10% FDR threshold was used.

### 4.6. *In vitro* model of gut permeability

Monolayers of Madin-Darby Canine cocker spaniel kidney (MDCK, Sigma-Aldrich) were cultured in on Millicell 0.4 µm PTFE Transwell inserts in a 24-well plate. Only passages between 17 and 22 were used for this study. The integrity of individual monolayers was assessed by measuring trans-epithelial electric resistance (TEER) using an Epithelial volt/ohm meter (EVOM). Fecal water was prepared and diluted in PBS to get a standard protein concentration of 300 ug per 200 μL of PBS+fecal water. Monolayers were treated with fecal water from samples of patients enrolled in the study to assess the effect on TEER. Hanks’ Balance Sal Solution (HBSS) was used as negative control, while *Enterococcus faecalis* spent medium as positive control. Further details on the model *in vitro* are provided in Supplementary Material.

### 4.7. Measurement of cytokines and markers of gut barrier integrity

The levels of interferon-gamma (IFN-γ), interleukin (IL) 1β, IL-2, IL-4, IL-6, IL-8, IL-10, IL-12p70, IL-13, and tumor necrosis factor-alpha (TNF-α) were measured in serum and fecal samples using the V-plex Proinflammatory Panel 1 (Meso scale discovery; MSD; Maryland, United States). The level of serum fatty acid-binding protein 2 (FABP-2) was measured using a Human FABP2/I-FABP Quantikine Enzyme-Linked immunosorbent Assay Kit (ELISA, R&D, USA).

## Supplementary Material

MASLD_revision_supplementary clean.docx

## Data Availability

The authors confirm that the data supporting the findings of this study are available within the article and its supplementary materials.
