# Supplementary material for: Disruption of gut barrier integrity and host–microbiome interactions underlie MASLD severity in patients with type-2 diabetes mellitus
Source: Gut Microbes. 2024 Jan 18;16(1):2304157. doi: 10.1080/19490976.2024.2304157 (PMC10798360; doi:10.1080/19490976.2024.2304157)
Supplement: MASLD_revision_supplementary clean.docx [file KGMI_A_2304157_SM3446.docx]

**SUPPLEMENTARY MATERIAL**

**METHODS**

***Metataxonomics***

Paired-end sequencing data were demultiplexed, barcodes removed and FASTQ files generated using Illumina software BCL Convert. DADA2 R-package (version 1.22.0) was used to process the fastq files. In short, 19 nucleotides from the forward and reverse primers were removed from the sequences and poor-quality reads (quality score < 30) were trimmed at position 250 - for the forward reads - and 150 - for the reverse reads-. After trimming step, the maximum number of expected errors in a read was set to 2 to discard reads with unique sequences. Sequence merging, denoising, removing of chimeras, and inferred sample sequences were processed using DADA2 default settings. Taxonomy of the amplicon sequence variants (ASV) was assigned using SILVA v132 reference database ([http://www.arb-silva.de](http://www.arb-silva.de)); accessed 28th May 2019 ).

***Ultra-high performance liquid chromatography-mass spectrometry bile acid profiling***

Study reference (SR) samples were prepared through the pooling of equal volumes of each prepared study sample and run interspersed with the study samples, to serve as quality control of the data acquisition and monitor assay performance. SR samples were also spiked with mixtures of 81 bile acid (BA) standards to determine retention time windows (regions of interest) for subsequent BA annotation. In addition, for assessment of linearity of analyte response ^1^, a series of SR sample dilutions was created by diluting with ice-cold LC-MS grade methanol to the concentrations of 100%, 80%, 60%, 40%, 20%, 10%, 1% and analyzed at the start and end of each set of sample analyses. Waters. RAW LC-MS data files were converted to .mzML format using Proteowizard msconvert ^2^, with removal of signals with less than 100 ion counts. Retention times and m/z values for each spiked BA standard were used to annotate and integrate their peaks relative abundance using peakPantheR package ^3^. To adjust for spectra intensity decay along the run, each feature was divided by a LOWESS curve fitted on the SR samples’ intensity.

Drift-correction and feature filtering were performed with the nPYc-Toolbox ^4^. Features with a coefficient of variation greater than 30% in normalised SR samples, with a Pearson correlation with dilution factor below 0.7 (estimated using a dilution series of pooled SR samples), or below limit of detection (LOD) in more than 20% of study samples were discarded. Features were log-transformed and zeroes imputed using impute. QRILC from the imputeLCMD R package. For statistical analyses, features were also mean-centered.

***Statistical analysis***

Sample matching

For each acquired omics dataset, matching was done on the analysed samples. Specifically, a Euclidean distance matrix was first created using the unimputed variables. Categorical variables were first converted into integers and continuous variables were untransformed, to implicitly have more weight when computing the distances (see **Supplementary Table 1** for the list of variables used for the matching). For each sample from a patient with either no MASLD or MASLD with fibrosis, a MASLD without fibrosis sample with the minimum distance value was selected. Sample size across groups was not artificially balanced by iteratively removing each match from the selection population.

***In-vitro model of gut permeability***

Frozen vials of Madin-Darby Canine cocker spaniel kidney (MDCK, Sigma-Aldrich) were stored in liquid nitrogen until use. MDCK were thawed and cultured with complete media (modified Dulbecco’s Modified Eagle Medium (DMEM) 500 ml + 1ml Plasmocin + 50ml Fetal bovine serum + 5ml 200mM L-glutamine) at 37.0°C, 5% CO_2_ until this reached confluence (typically after 48-72 hours). Aliquots of 1-2 x 10^5^ MDCK cells were cultured in on Millicell 0.4 µm PTFE Transwell inserts (0.3 cm^2^ surface area, Merck KGaA, Darmstadt, Germany) in a 24-well plate. Cultured cells were maintained at 37°C, 5% CO_2_, for 72-96 hours to allow for monolayer formation, while culture media was changed every day. As some level of variability from different passages was previously described^8^, only passages between 17 and 22 were used for this study. The integrity of individual monolayers was assessed by measuring trans-epithelial electric resistance (TEER) using an Epithelial volt/ohm meter (EVOM). Monolayers were considered intact when TEER measurements were stable after at least 2 days from seeding. Monolayers with TEER values < 150 Ωcm^2^ after 72 hours were not considered intact and were excluded from use.

Faecal water (FW) was prepared from phosphate-buffered solution (PBS) and stool samples in order to obtain a standard protein concentration of 300 μg total protein per 200 μL of PBS+FW solution. *Enterococcus faecalis* (*E. faecalis*) was chosen as positive control, given its distinctive proteolytic activity through the production of specific gelatinases and serine proteases^9^. At time zero, Hanks' Balance Sal Solution (HBSS) from the apical compartment of each well was removed and replaced by equal volume of either PBS (as negative control), *E.faecalis* spent medium (as positive control) or FW derived from the faecal samples of patients enrolled in the study. Once PBS, *E. faecalis* spent medium and FW were added to the wells, TEER was then measured at different time points (5, 30, 90, 120 min and 24 hours). Experiments with FW were performed in triplicates and were also replicated with the addition of a commercial bacterial protease inhibitor cocktail (Merck Life Science UK Limited, UK).

**SUPPLEMENTARY FIGURES**


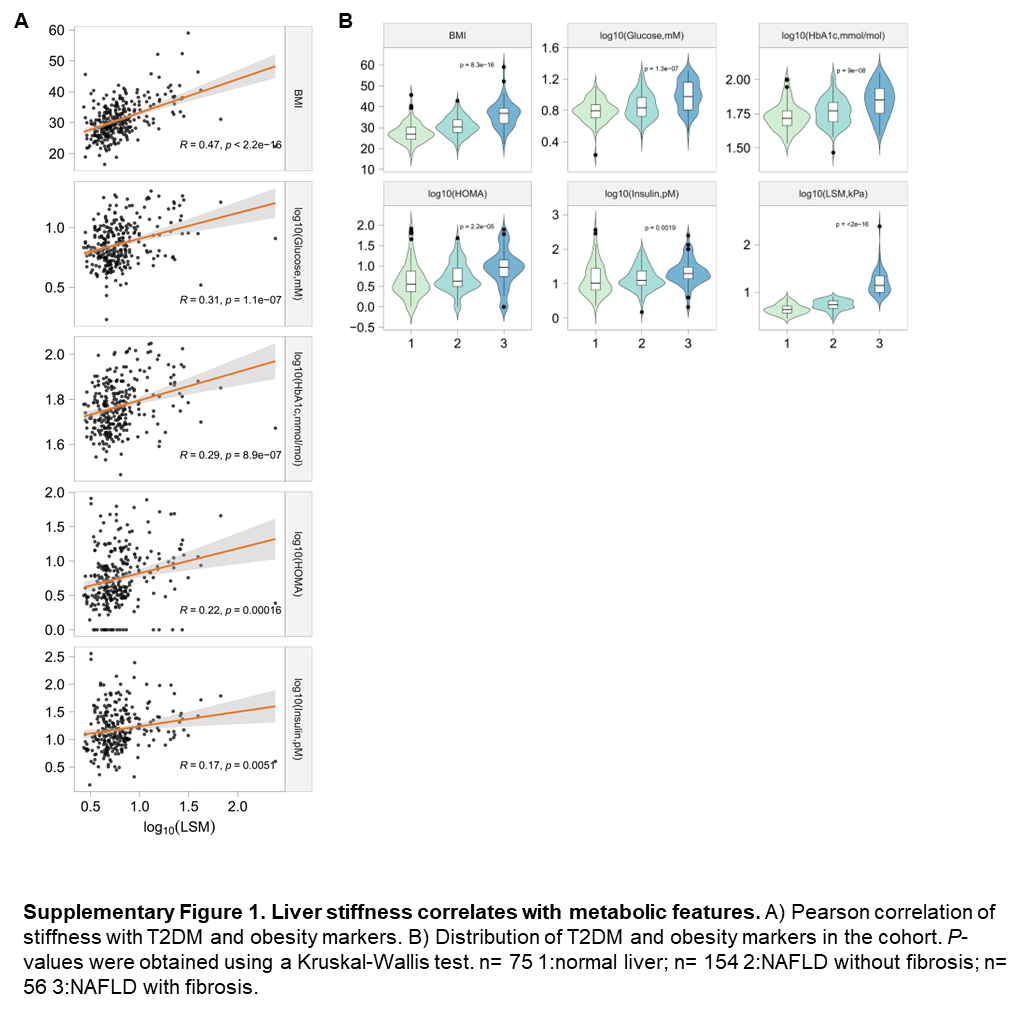


**Supplementary Figure 1. Liver stiffness correlated with metabolic features.** A) Pearson correlation of stiffness with T2DM and obesity markers. B) Distribution of T2DM and obesity markers in the cohort. P-values were obtained using a Kruskal-Wallis test. N=75 “1”: normal liver; n=154 “2”: MASLD without fibrosis; n=56 “3”: MASLD with fibrosis.


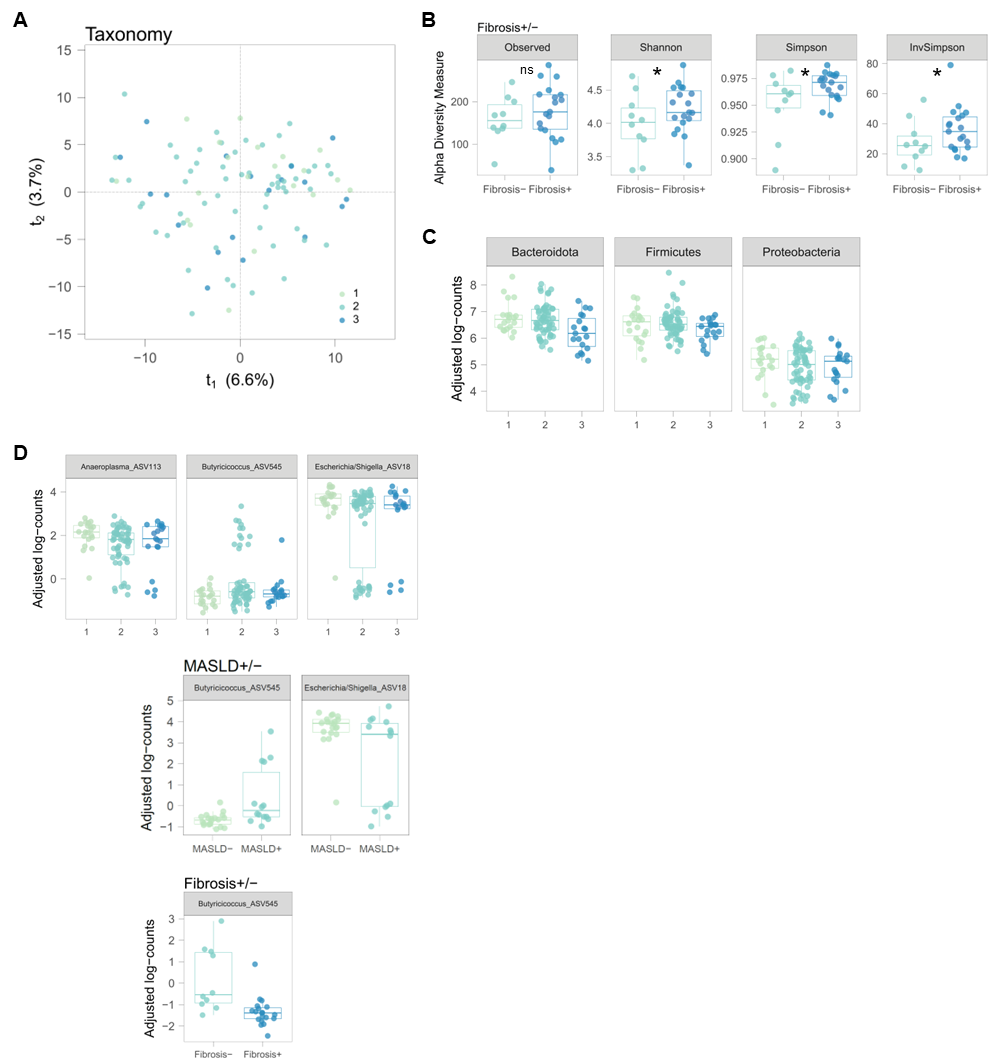


**Supplementary Figure 2. Taxonomy unmatched and matched analysis.** A) Principal Component Analysis (PCA) of Aitchison distance beta diversity (N=97; 20 “1”; 58 “2”; 19 “3”). B) Alpha diversity measurements in Fibrosis +/- subsets (n=10 Fibrosis-; n= 19 Fibrosis+). Significance was determined using a likelihood ratio test of two nested mixed effects models with or without the group variable. C) Significantly abundant phyla and D) ASVs in the unmatched dataset, determined with ANCOM-BC. N=97 (20 “1”; 58 “2”; 19 “3”). Values are the log-transformed counts adjusted by sampling fraction determined by the ANCOM-BC algorithm.

Supp


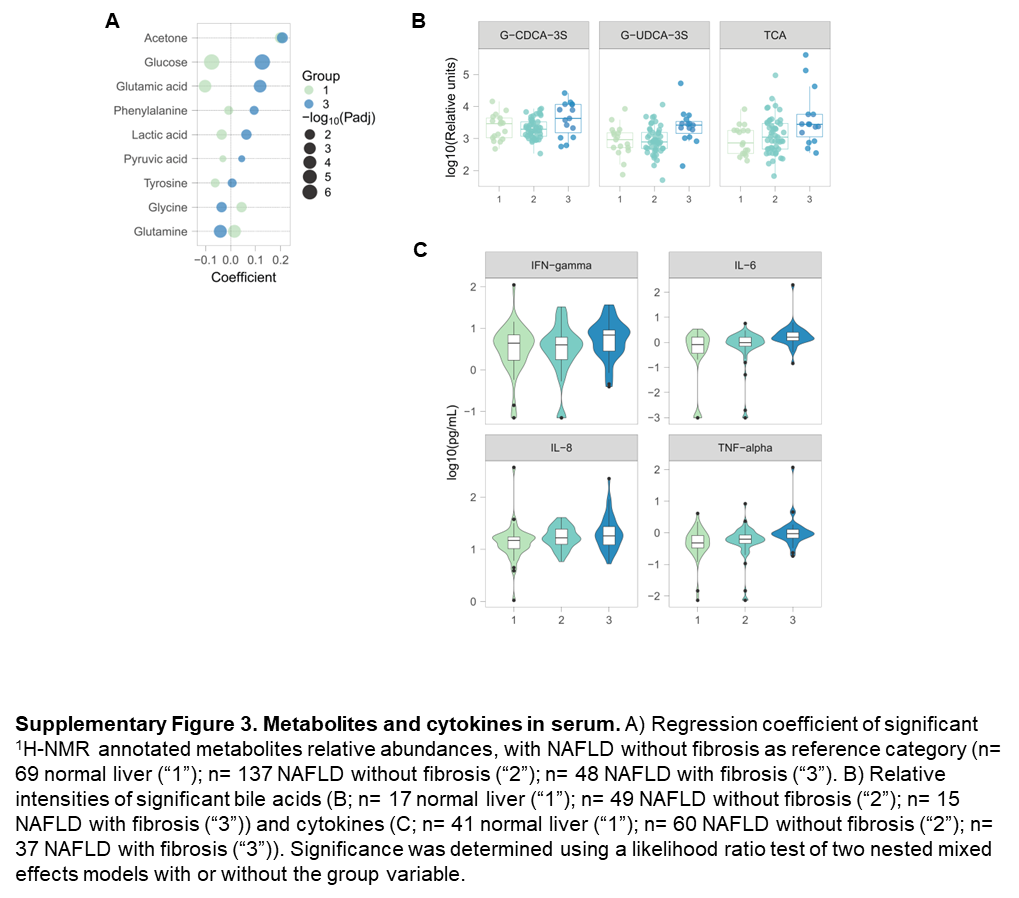


**Supplementary Figure 3. Metabolites and cytokines in serum.** A) Regression coefficient of significant ^1^H-NMR annotated metabolites relative abundances, with MASLD without fibrosis as reference category (n=69 normal liver (“1”); n=137 MASLD, without fibrosis (“2”); n=48 MASLD with fibrosis (“3”). B) Relative intensities of significant bile acids (n=17 normal liver (“1”), n=49 MASLD without fibrosis (“2”), n=15 MASLD with fibrosis (“3”) and C) cytokines (n=41 normal liver (“1”); n=60 MASLD without fibrosis (“2”); n=37 MASLD with fibrosis (“3”)). Significance was determined using a likelihood ratio test of two nested mixed effect models with or without the group variable.


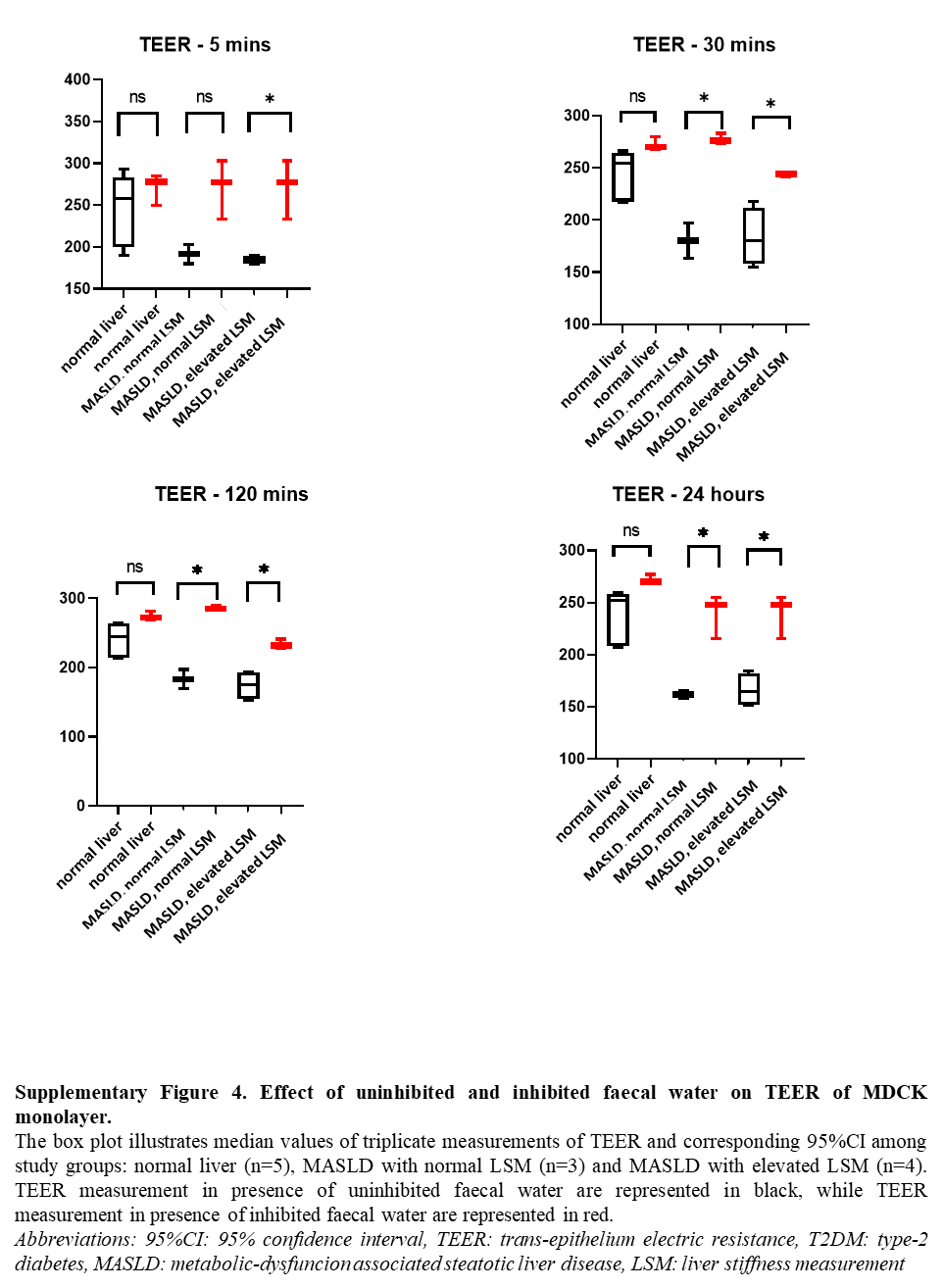


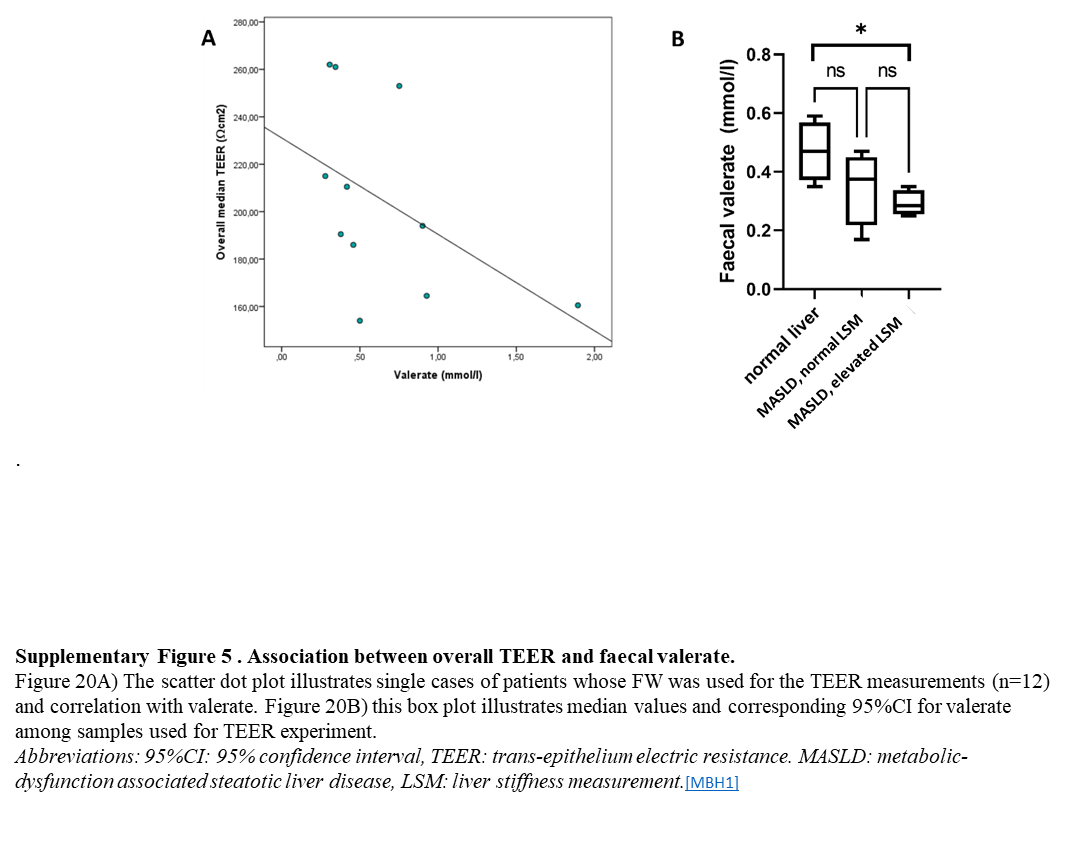


**Supplementary Table 1. Variables used to construct the matched datasets**


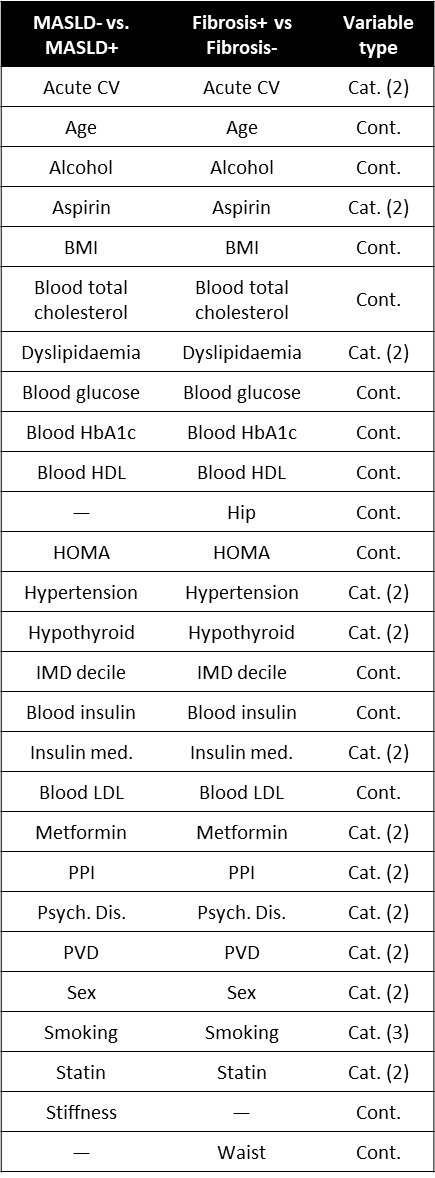


**Supplementary table 2. Clinical characteristics of the patients whose fecal samples were used for TEER analysis.**

|  | **Normal liver**  **N=5** | **MASLD, normal LSM**  **N=3** | **MASLD, elevated LSM**  **N=4** |
| --- | --- | --- | --- |
|  | **Median (IQR)** | **Median (IQR)** | **Median (IQR)** |
| **Age, *years*** | 59 (52-61) | 61 (54-65) | 62 (60-67) |
| **Waist circum, *cm*** | 96 (73-103) | 105 (99-13) | 121 (113-128) |
| **BMI, *kg/m^2^*** | 26.7 (19.4-30.6) | 30.3 (27.6-33.6) | 40.3 (38.2-44) |
| **PLT, *x 10^9^/µL*** | 225 (186-266) | 255 (215-300) | 255 (197-328) |
| **ALT, *IU/L*** | 18 (15-41) | 30 (22-43) | 22 (19-51) |
| **AST, *IU/L*** | 22 (22-28) | 26 (22-32) | 29 (23-45) |
| **GGT, *IU/L*** | 17 (15-56) | 27 (19-39) | 32 (19-78) |
| **Total Cholesterol, *mmol/l*** | 3.8 (3.6-4) | 4.1 (3.5-4.8) | 4 (3.6-5.3) |
| **Ferritin, *ng/ml*** | 62 (24-76) | 82 (36-124) | 129 (54-139) |
| **CAP score, *dB/m*** | 208 (175-244) | 324 (300-394) | 356 (332-391) |
| **LSM, *kPa*** | 4.4 (4.1-6.3) | 5.4 (4.5-6.4) | 18.2 (11-22.2) |
| **Diabetes characteristics** | | | |
|  | **Median (IQR)** | **Median (IQR)** | **Median (IQR)** |
| **Fasting glucose, *mmol/l*** | 4.4 (4.3-8.4) | 6.7 (5.2-9.2) | 5.9 (5.1-8.1) |
| **HbA1c, *mmol/mol*** | 57 (55-61) | 59 (49-68) | 67 (44-74) |
| **Homa index** | 3.7 (0.5-3.8) | 3.3 (2.1-8.4) | 2.3 (0.4-8.8) |

1. Sands CJ, Gomez-Romero M, Correia G, et al. Representing the Metabolome with High Fidelity: Range and Response as Quality Control Factors in LC-MS-Based Global Profiling. *Anal Chem* 2021;93(4):1924-33. doi: 10.1021/acs.analchem.0c03848 [published Online First: 20210115]

2. Chambers MC, Maclean B, Burke R, et al. A cross-platform toolkit for mass spectrometry and proteomics. *Nat Biotechnol* 2012;30(10):918-20. doi: 10.1038/nbt.2377

3. Wolfer AM, Correia GDS, Sands CJ, et al. peakPantheR, an R package for large-scale targeted extraction and integration of annotated metabolic features in LC-MS profiling datasets. *Bioinformatics* 2021;37(24):4886-8. doi: 10.1093/bioinformatics/btab433 [published Online First: 20210614]

4. Sands CJ, Wolfer AM, Correia GDS, et al. The nPYc-Toolbox, a Python module for the pre-processing, quality-control and analysis of metabolic profiling datasets. *Bioinformatics* 2019;35(24):5359-60. doi: 10.1093/bioinformatics/btz566

5. Martinez-Gili L, McDonald JAK, Liu Z, et al. Understanding the mechanisms of efficacy of fecal microbiota transplant in treating recurrent Clostridioides difficile infection and beyond: the contribution of gut microbial-derived metabolites. *Gut Microbes* 2020;12(1):1810531. doi: 10.1080/19490976.2020.1810531

6. Lin H, Peddada SD. Analysis of compositions of microbiomes with bias correction. *Nat Commun* 2020;11(1):3514. doi: 10.1038/s41467-020-17041-7 [published Online First: 20200714]

7. Martinez-Gili L, Pechlivanis A, McDonald JAK, et al. Bacterial and metabolic phenotypes associated with inadequate response to ursodeoxycholic acid treatment in primary biliary cholangitis. *Gut Microbes* 2023;15(1):2208501. doi: 10.1080/19490976.2023.2208501

8. Furuse M, Tsukita S. Claudins in occluding junctions of humans and flies. *Trends in cell biology* 2006;16(4):181-8. doi: 10.1016/j.tcb.2006.02.006 [published Online First: 2006/03/16]

9. Nesuta O, Budesinsky M, Hadravova R, et al. How proteases from Enterococcus faecalis contribute to its resistance to short alpha-helical antimicrobial peptides. *Pathog Dis* 2017;75(7) doi: 10.1093/femspd/ftx091 [published Online First: 2017/08/24]
